# The Charter of the Medical Society of the State of New York

**Published:** 1903-01

**Authors:** 


**A Monthly Review of Medicine and Surgery.**

*EDITOR:*

WILLIAM WARREN POTTER, M. D.

All communications, whether of a literary or business nature, books for review and exchanges should be addressed to the editor: 284 FRANKLIN STREET, BUFFALO, N. Y.

VOL. XLII.—LVIII. JANUARY, 1903. NO. 6.

### The Charter of the Medical Society of the State of New York.

IN THE December issue of the JOURNAL we gave editorially our first impressions of the plan, proposed by the committees of conference, "to charter the Medical Society of the State of New York." We now offer some supplementary impressions upon that unique proposition the result of a further study of the proposed bill.

Careful reading of that peculiar document reveals additional evidence that it was written in a spirit distinctly antagonistic to the interests of the state society; or, if not, its verbiage is unfortunate.

For example, we are told, in the preamble and in section six, that the state society was "organised" and "founded" in 1806, and that the state association was "incorporated in 1884, and chartered in 1900." That there may be no uncertainty as to the fact that the state society up to this time has had, in the mind of the author of this bill, neither incorporation nor charter, is made as plain as words can make it in the next sentence of the preamble giving the purpose of the law in its title—namely, "an act, to charter the Medical Society of the State of New York," and section one adds the impressive words "There shall be established by the physicians named in section six of this act an organisation styled 'The Medical Society of the State of New York.'" We are unwilling to believe that such misrepresentation is studied and intentional, but rather that it is accidental and thoughtless.

It is to be noted, however, that the society sought to be founded by the proposed bill, is most undemocratic in composition and effect. Section five provides for "the superintendence and management of 'the Medical Society of the State of New York' by 'a body known and styled the council and Fellows,'" and gives to this body, which possibly may be very small in numerical aggregate, the minimum number not being named, not only the broad general powers of "superintendence and management," but also the specific "power to make and prescribe by-laws that shall govern its officers, council, fellows, and members; to establish the conditions of admission, dismission and expulsion of members; to determine the amount of the annual dues and also to impose assessments from time to time on its members; to collect such dues and assessments by suit or otherwise, and to receive, hold, invest, or otherwise dispose of all moneys or other property belonging to the said Medical Society of the State of New York, and in general to make such by-laws, rules and regulations for the proper government of the society and of its branches and subordinate county societies, etc., etc."

We regard this plan as undemocratic, and unAmerican if not intolerably autocratic. Moreover, it is, in our view, undignified to "superintend" and "manage" a great society of scientific physicians, as if it were a steel plant or other commercial enterprise.

Again, it is to be remarked that the proposed bill, if enacted would abolish the state and county societies, and possibly create an interval of considerable and indefinite duration, in which the homeopathic and eclectic state and county societies would be the only organised medical societies in the state, and would leave to the former of these the title of seniority by nearly half a century —namely, from 1857 to 1903.

Still again, the proposed bill is unfair and unjust to the state society in that it makes members of the entire association, which is in the nature of a mass meeting organisation, whereas the state society is a delegate body.

The proposed bill is liable to further objection in that it contemplates a business corporation, law practice, and life insurance —all worthy and possibly gainful pursuits, but not the high single aim and level of the preamble of 1806.

But it is not likely that the representatives of the state society have assented to the proposed bill, because their instructions did not contemplate such radical action. An attentive study of the president's inaugural address, in which the conference committee was suggested, and of the report of the committee to whom the address was referred, in which the action taken by the society was advised and through which it was finally authorised, fails to find the slightest intimation that the state society contemplated repudiation of its birthright. The title of this division of the inaugural address, "The relations of the Medical Society of the State of New York to the National Profession," clearly points out the object of the committee advised, and the following sentences just as clearly indicate why it seemed to the president that the work should be undertaken at this time: "because of the encouragement received by the reorganisation upon a broad platform of the American Medical Association."

This reorganisation had brought the organic law of the national body into harmony with that of the state society, hence affiliation was possible and, of course, desirable.

The object and method of the committee were plainly laid down in the resolution appointing it which goes on to say:

"In the event of failure" of the preliminary negotiations "the committee appointed by the Medical Society of the State of New York shall have full power . . . to represent this society before the American Medical Association and the secretary of this society shall . . . provide the individual members with credentials to represent this society in the American Medical Association."

It would seem from the above that the real, the important, work of our committee is yet to be undertaken.

Finally, we think this proposed bill objectionable to the state society as a basis of medical organisation in that it is wanting in maturity, breadth, catholicity and simplicity; moreover, it may not be appealed to as non-sectarian as may the law of 1806.

We all know and acknowledge with pleasure that the state association membership includes men of eminent personality, of broad and noble aspirations for our profession and its higher ideals, but we also remember that the history of that association shows that it has had human guidance and that its purposes have not always been free from sectarian tendencies. That these tendencies should find expression in its organic law is but natural, but that its organic law, scarcely three years old, should be thought superior to that of the state society, representing the wisdom and experience of a century, is incredible.

We may be pardoned we trust for pointing out in the published proceedings of the state association a virtual ultimatum offered and passed at its last meeting, that will be found on another page, which indicates to our mind that the times are not propitious for successful negotiations in this direction; and we are glad that an alternative anticipating such a situation was prepared in the instructions to our committee.

This alternative, the sending of a committee asking membership in the American Medical Association upon the credentials of the Medical Society of the State of New York, seems to us to be a comparatively simple and easy way of testing the temper of the national body which, as our memory serves, is or was the real party in controversy with our state society. The American Association itself having swept away the barriers of difference there should be no reason why it would not deal directly with our ambassadors. Why not, if we have anything to say, try the American method of diplomacy and make a plain statement of our case at headquarters? At least, this plan would appear to the average observer as worthy of trial.

